# Cereal Consumption among Subjects with Celiac Disease: A Snapshot for Nutritional Considerations

**DOI:** 10.3390/nu9040396

**Published:** 2017-04-18

**Authors:** Francesco Valitutti, Donatella Iorfida, Caterina Anania, Chiara Maria Trovato, Monica Montuori, Salvatore Cucchiara, Carlo Catassi

**Affiliations:** 1 Paediatric Gastroenterology and Liver Unit, Sapienza University of Rome, 00161 Rome, Italy; francesco.valitutti@uniroma1it (F.V.); donatella.iorfida@uniroma1.it (D.I.); caterina.anania@uniroma1.it (C.A.); chiaramaria.trovato@uniroma1.it (C.M.T.); monica.montuori@uniroma1.it (M.M.); salvatore.cucchiara@uniroma1.it (S.C.); 2 Department of Pediatrics, Università Politecnica delle Marche, 60123 Ancona, Italy

**Keywords:** celiac disease, cereal-based products, gluten-free diet, nutrition, dietary survey

## Abstract

Background: To our knowledge no study has focused on the pattern of cereal-based products (CBP) consumption among people with celiac disease (CD). Our study aimed at evaluating the dietary intake of CBP among patients with CD and comparing it with a control population. Methods: Eighty-two volunteers with CD and 77 non-CD volunteers enrolled throughout Italy were asked to register their consumption of CBP on specific diaries for three days. Results: CD patients’ median three-day intake of biscuits and crackers was higher compared to controls (65.8 g vs. 22.7 g and 44.7 g vs. 10.6 g, *p* < 0.05 respectively, Mann–Whitney test). A significant difference was observed also comparing the two groups for median three-day bread consumption, with the CD group consuming less bread than controls (109.5 g vs. 150.7 g, *p* < 0.05, Mann–Whitney test). When assessing regional and gender-related CBP consumption patterns, significantly higher rice consumption was found among CD women from Northern Italy compared to CD women from Central and Southern Italy (*p* = 0.006 and *p* = 0.002 respectively, Fisher’s exact test). No other significant differences were observed. Conclusions: Our results provide a snapshot of the overall consumption of CBP among Italian subjects with CD. Altogether, these data show that, despite minor differences, dietary consumption of CBP among CD patients is similar to the general population.

## 1. Introduction

Celiac disease (CD) is an immune-mediated systemic disorder triggered by the ingestion of gluten and related prolamines in genetically susceptible individuals [1].

The prevalence of CD varies from 4.5% among first-degree relatives of CD-affected patients to 0.5%–1.0% in the general population [2,3] and is increasing over time [4,5]. The frequency increase is partially related to improved diagnostic rates, as a result of increased awareness by health care professionals and the easy access to reliable serologic testing [6]; however, a true increase of prevalence has also been identified and this could be attributable to still elusive and hypothetical environmental triggers [7].

A strict gluten-free diet (GFD) is the only available treatment for CD: it relieves symptoms, achieves mucosal healing, and prevents disease complications [8,9]. However, many patients find it difficult to comply with the diet [10]. GFD consists of a combination of naturally occurring gluten-free foods (e.g., gluten-free cereals, fruit, vegetables, unprocessed meat, fish) and specially manufactured gluten-free substitutes of wheat-based foods such as pasta, bread, cereals, crackers, and snack foods, in which wheat flour is replaced by gluten-free flour [11]. 

Although conceptually simple, the changes imposed on the daily diet are substantial and have a profound effect on the dietary habits of CD patients and their families. There are many barriers to the GFD, including the availability, cost, and safety of gluten-free food and gluten cross-contamination [12,13].

Regular follow-up visits are needed to assess compliance, as well as to ensure nutritional adequacy and prevent overnutrition while adhering to a lifelong GFD [14]. Patients pursuing their everyday gluten avoidance might overlook the importance of eating a balanced diet. The GFD definitely restricts food choices and has been identified as a possible cause of inadequate nutrient intake [15,16,17]. Most commercial gluten-free foods are alternatives to traditional wheat-based products and it has often been stated that gluten-free processed foods are less healthy than their gluten-containing counterparts, due to relatively higher content of fat, sugar, and salt [18,19], but current data on this issue are scarce [20]. 

To our knowledge no study has focused on the pattern of cereal-based products (CBP) consumption among people with CD. The present study aimed at assessing the dietary intake of CBP among CD patients compared to a group of healthy controls. 

## 2. Materials and Methods 

Eighty-two volunteers with biopsy-proven CD and 77 non-CD healthy volunteers were enrolled in this study through our internal patient database (Rome and Ancona) and via Italian Celiac Society social media (magazine, newsletters, websites, email, Facebook and Twitter) from November 2011 to March 2013. Controls were not significantly different in terms of age or gender and were chosen among friends or colleagues of CD patients. General exclusion criteria for both CD and control group were a diagnosis of diabetes mellitus (both type-1 and type-2), anorexia, bulimia, food allergy or being on a restricted diet for other medical/ethical/religious reasons (including non-celiac gluten sensitivity among controls). A specific exclusion criterion for the CD group was a CD diagnosis within one year from the recruitment time. Being a family member of CD patients was an additional exclusion criterion for the control group.

Demographics of study participants are summarized in Table 1. 

All subjects received oral and written information about the nature and purpose of the survey and all of them formally consented to the study. This study was approved by the Ethical Committee of the Politecnica delle Marche University of Ancona, Italy (N533/Oct 2011).

Dietary intakes of CD patients and controls were collected using a three-day food diary. All participants recorded their consumption of CBP for 3 consecutive days using a specific dietary diary. 

A list of the food item to be registered was provided to the study subjects. Alongside with this list, an example sheet guiding participant to correctly fill the boxes was also given. Main meals and additional snacks between main meals were represented as specific boxes to fill on the data sheet. During the days of the survey, participants were advised to maintain their usual eating habits. Volunteers registered ingested CBP in detail, specifying the type, brand, and weight by means of a digital scale. For each “out of home” meal, instead of weighing they had been allowed to use a photo atlas to determine the weight of food consumed (photo atlas of food portions, Istituto Scotti Bassani 2008). “Out of home” meals accounted for 5.1% of all recorded meals. 

The consumption over three days of sweet pastries (processed plum cakes, muffins and croissants), bread, pasta, pizza, homemade cakes, biscuits, crackers, breakfast cereals, breadcrumbs, and other cereals of the CD group was compared with that of the control group. 

Data were expressed as median daily and three-day consumption of each CBP category. The Mann–Whitney test was used for comparisons. For regional subgroup analysis, data were expressed as the median three-day consumption or percentage of consumption (consumers vs. non-consumers) during the three days of the survey. The Mann–Whitney test or Fisher’s exact test were used to compare subgroup data as appropriate. Statistical analysis was performed by Prism software version 5.00 (GraphPad, San Diego, CA, USA). 

## 3. Results

**Figure 1:**
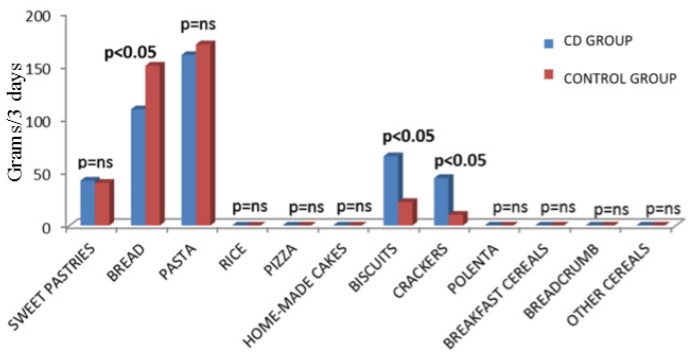
Median three-day consumption of cereal-based products in 82 celiac patients and 77 healthy controls. Data are expressed in grams.

The median three-day consumption of CBP of CD patients and controls is represented in Figure 1. Overall comparisons between the two groups are also summarized in Table 2. As far as the median three-day consumption of different CBP, CD patients’ intake of bread was lower compared to controls (109.5 g vs. 150.7 g, *p* < 0.05, Mann–Whitney test). Conversely, a significant higher median three-day consumption of biscuits (65.8 g vs. 22.7 g, *p* < 0.05, Mann–Whitney test) and crackers (44.7 g vs. 10.6 g, *p* < 0.05, Mann–Whitney test) was found among CD subjects compared to controls. As regards the consumption of all the other CBP analyzed, no significant differences were observed between the CD and the control group.

Among males, a higher daily intake of biscuits (41.2 g vs. 0 g, *p* < 0.05, Mann–Whitney test) and a higher daily intake of crackers (13.8 g vs. 0 g, *p* < 0.05, Mann–Whitney test) were found among CD subjects. 

On the other hand, female subgroup analysis showed a reduced daily consumption of bread among CD women compared to controls (33.2 g vs. 50.6 g, *p* < 0.05, Mann–Whitney test). 

Table 3 and Table 4 show consumption patterns (expressed in grams) of the two gender subgroups. 

When assessing regional CBP consumption pattern, a significantly higher frequency in rice consumption was found among CD women from Northern Italy (63%) compared to those from Central (16%) and Southern Italy (8%) (*p* = 0.006 and *p* = 0.002 respectively, Fisher’s exact test). No other significant differences were noticed with regard to regional and gender CBP consumption. (supplementary figures) 

**Figure 2:**
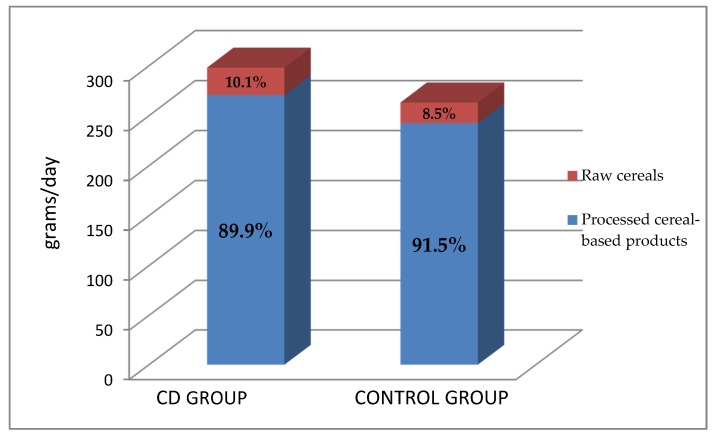
Daily cereal-based product consumption pattern in CD patients and controls according to food processing: raw cereals (rice, polenta, other cereals) vs. processed cereal-based products (sweet pastries, bread, pasta, homemade cakes, biscuits, crackers, breakfast cereals, and breadcrumbs).

**Figure 3:**
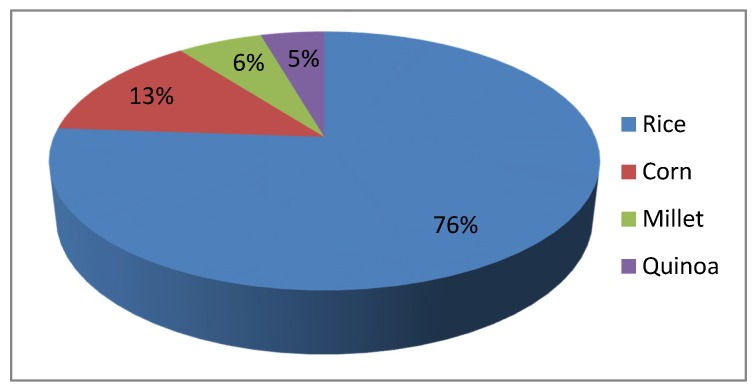
Raw cereal consumption of CD patients.

**Figure 4:**
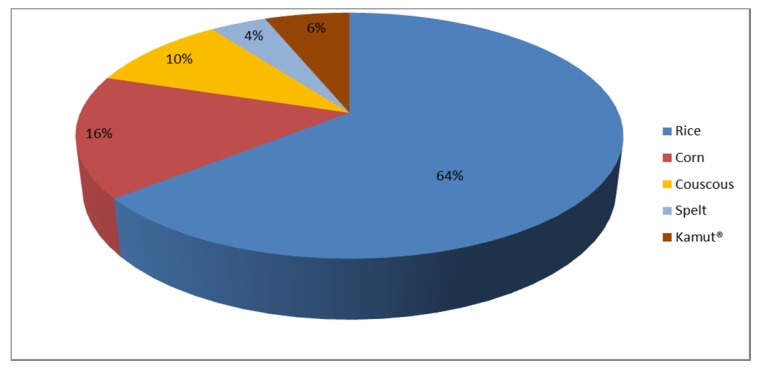
Raw cereal consumption of the control group.

When comparing daily CBP consumption pattern in CD and control groups according to food processing (raw cereals vs. processed cereal-based products), we also found a similar distribution between CD and control consumption patterns (Figure 2). We found that rice and corn were the most consumed naturally GF cereals in the CD group, followed by millet and the pseudo-cereal quinoa. Conversely, as regards naturally gluten-containing cereals, controls consumed mostly wheat couscous, followed by kamut^®^ and spelt. Raw cereal consumption in both group is represented in Figure 3 and Figure 4. 

## 4. Discussion

Although considerable scientific progress has been made in CD epidemiology, pathogenesis and diagnosis, a strict gluten-free diet is still the only mainstay of treatment for this disorder, since the first description made by the Dutch physician Dicke in the early 1950s [21].

GFD entails avoidance of the gluten-containing grains wheat, rye, and barley, whereas rice, oats, buckwheat, corn, millet, and quinoa are included [22]. Although the availability of gluten-free foods is still limited to some extent, recent years have witnessed a tremendous increase for the GF market. As it was recently pointed out by a market research report, the GF products market is experiencing a double-digit growth on a yearly basis [23]. This means that the GF product market represents one of the most prosperous markets in the field of food and beverages in the next future. Eating and baking gluten-free has become easier in recent years, with increases in the number and quality of gluten-free products that are available on the Internet and in many stores today, albeit at a greater expense compared to gluten-containing foods [11]. CD patients, at least in some Western countries, receive financial assistance to compensate for this higher cost, and this support has also been shown to enhance compliance rates [24]. Consumers can purchase packaged gluten-free breads, buns, rolls, pizza crusts, donuts, pastas, pretzels, cereals, and desserts. Baking mixes and flours are also available. The availability of gluten-free products increases patients’ food choices, improves diet variety, while allowing patients to feel “normal” when eating with others [14], and improving also dietary compliance [24]. 

Our study clearly showed that patients with CD tend to reproduce the pattern of CBP consumption of healthy controls, as shown by the marked overlapping of the quantities of different products consumed by the two groups. This is an interesting and somewhat unexpected finding given that all CD patients at diagnosis are usually encouraged to increase their intake of naturally gluten-free cereals (or pseudo-cereals) such as rice, corn, and buckwheat. In our opinion, difficulties in changing structured eating behavior, poor diffusion of naturally GF cereals, and fear of gluten contamination are the most likely explanation of this finding. 

By comparing CD patients and controls, significant differences were observed only as regards the consumption of bread, biscuits, and crackers. Biscuit and cracker consumption was higher among CD patients than among controls, while bread intake was lower in the CD group compared to controls. These data might underline that CD consumers rely more on biscuits and crackers as sources of carbohydrates than the healthy population. This should drive both dieticians’ and physicians’ thoughtful considerations of the nutritional adequacy of the GF diet, since biscuits show a tendency to a higher glycemic index. As the lack of gluten itself may imply an overall increase of the glycemic index [25], these data need a careful consideration due to their possible metabolic long-term consequences. Moreover, GFD seems to increase the risk of excess weight and obesity because of the nutritional imbalance and hypercaloric content of commercial gluten-free foods [26]. Additionally, the content of highly absorbable sugars of the majority of these products can contribute to the occurrence of hyperinsulinemia and insulin-resistance with overweight, thus leading to an increased risk of metabolic syndrome [27]. Healthy food choices include a significant amount of dietary fiber, low amounts of simple carbohydrates, and thus a lower glycemic index [28]. Consequently, CD individuals may improve the overall nutritional quality of their diet by increasing the intake of fiber [20]. 

The CD group consumed less bread than controls. This trend could have two conceivable interpretations: firstly, this different consumer choice could be possibly related to a current gap of palatability with gluten-containing bread; secondly, a limited availability of freshly baked GF bread could also limit the bread consumption and explain this result. 

In our CD group, bread was prepared with several flour mixes available on the market. The main components of these gluten-free flours were rice or maize, usually followed by rice starch and potato starch.

The analysis of subgroup intakes showed a significantly higher consumption of rice among CD women from Northern Italy when compared to CD women from Central and Southern Italy: we might interpret this finding as a regional difference, mirroring other data on regional rice consumption [29], albeit our control group did not show this pattern.

We are aware of certain drawbacks of our study. Firstly, the population sample enrolled included a clear predominance of women (125 F:34 M), which goes beyond the expected higher CD prevalence in females than males (1.5–2:1). Women traditionally tend to show a greater interest and participate more in nutritional surveys [30,31]. Other potential limitations of this survey were the variable accuracy of self-reported data and possible dietary changes induced by the recording itself during the three days of the study [32,33]. We also acknowledge that individuals’ nutrient intake may fluctuate on a day-to-day basis due to a variety of factors, such as day of the week and season of the year [34], but we still trust that the three-day recording method is a useful tool for population studies. Another potential limitation of the survey might be the use of a “convenience sample” that included CD-patients being members of celiac support groups and/or subscribers to a national magazine on CD, who may not be representative of the general Italian celiac community. 

## 5. Conclusions

Physicians and dieticians alike should carefully review the dietary pattern of celiac patients at follow-up. 

This is the first study reporting on the dietary intake of CBP among CD patients. Our results snapshot overall consumption of CBP among Italian subjects with CD. We found a higher consumption of crackers and biscuits among CD subjects; this was counterbalanced by a lower bread consumption in the CD group. These results should encourage producers’ efforts in reducing glycemic index of GF products and in increasing the palatability of GF bread. However, altogether these data show that CD patients’ diet generally reproduce, despite minor differences, the eating behavior of the general population. 

## Figures and Tables

**Table 1:** Demographics of celiac and control participants.

Demographic Data	Celiac Subjects	Control Subjects
M/F ratio	14/68	20/57
Median age (range)	37 years (18–67)	37 years (18–68)
Median weight (range)	64.2 kg (47.2–88.6)	69.3 kg (52.8–90.1)
Median height (range)	168.9 cm (148.3–189.5)	166.1 cm (146.8–192.0)

**Table 2:** Consumption of cereal-based products in celiac disease (CD) subjects compared to controls. Data are expressed as median three-day consumption and inter-quartile range. Mann–Whitney test was used to compare group data.

Cereal-Based Products	Celiac Population	Control Population	Statistics
Sweet Pastries	42.1 g (0–146)	40.2 g (0–100)	*p* = ns
Bread	109.5 g (50–226)	150.7 g (80–234)	*p* < 0.05
Pasta	160.0 g (92–254)	173.3 g (105–232)	*p* = ns
Rice	0 g (0–99)	0 g (0-60)	*p* = ns
Pizza	0 g (0–200)	0 g (0–248)	*p* = ns
Homemade Cakes	0 g (0–0)	0 g (0–0)	*p* = ns
Biscuits	65.8 g (12–104)	22.7 g (0–92)	*p* < 0.05
Crackers	44.7 g (0–85)	10.6 g (0–65)	*p* < 0.05
Polenta	0 g (0–0)	0 g (0–0)	*p* = ns
Breakfast Cereals	0 g (0–57)	0 g (0–50)	*p* = ns
Breadcrumb	0 g (0–0)	0 g (0–0)	*p* = ns
Other Cereals	0 g (0–26)	0 g (0–43)	*p* = ns

**Table 3:** Median and inter-quartile range daily consumption of cereal-based products in CD patients and controls (males). Comparison was made by Mann–Whitney test.

Cereal-Based Products	CD Subjects (Males)	Controls (Males)	Statistics
Sweet Pastries	3.5 g (0–68.4)	20.8 g (0–63)	*p* = ns
Bread	69.1 g (47.2–125.6)	67.6 g (34.2–118.9)	*p* = ns
Pasta	58.4 g (48.2–150.9)	108.3 g (90.1–156.9)	*p* = ns
Rice	27.6 g (0–43.3)	0 g (0–0)	*p* = ns
Pizza	0 g (0–41.8)	0 g (0–23.6)	*p* = ns
Homemade Cakes	0 g (0–0)	0 g (0–0)	*p* = ns
Biscuits	41.2 g (18.7–65.1)	0 g (0–27.1)	*p* ˂ 0.05
Crackers	13.8 g (0–29.2)	0 g (0–0)	*p* ˂ 0.05
Polenta	0 g (0–0)	0 g (0–0)	*p* = ns
Breakfast Cereals	0 g (0–16.3)	0 g (0–58.5)	*p* = ns
Breadcrumb	0 g (0–0)	0 g (0–0)	*p* = ns
Other Cereals	0 g (0–0)	0 g (0–11.3)	*p* = ns

**Table 4:** Median and inter-quartile range daily consumption of cereal-based products among CD patients and controls (females). Comparison was made by Mann–Whitney test.

Cereal-Based Products	CD Subjects (Females)	Controls (Females)	Statistics
Sweet Pastries	16.6 g (0–44.7)	13.2 g (0–26.8)	*p* = ns
Bread	33.2 g (14.3–68.6)	50.6 g (27.2–80.4)	*p* ˂ 0.05
Pasta	53.3 g (31.0–84.6)	51.6 g (33.1–71.9)	*p* = ns
Rice	0 g (0–31.1)	0 g (0–23.6)	*p* = ns
Pizza	0 g (0–86.6)	0 g (0–83.2)	*p* = ns
Homemade Cakes	0 g (0–0)	0 g (0–0)	*p* = ns
Biscuits	19.6 g (0–33.0)	6.9 g (0–32.1)	*p* = ns
Crackers	14.8 g (0–29.6)	6.6 g (0–22.1)	*p* = ns
Polenta	0 g (0–0)	0 g (0–0)	*p* = ns
Breakfast Cereals	0 g (0–34.1)	0 g (0–16.4)	*p* = ns
Breadcrumb	0 g (0–2.5)	0 g (0–0)	*p* = ns
Other Cereals	0 g (0–21.4)	0 g (0–34.6)	*p* = ns
